# Molecular Dynamics Simulations Capture the Misfolding of the Bovine Prion Protein at Acidic pH

**DOI:** 10.3390/biom4010181

**Published:** 2014-02-10

**Authors:** Chin Jung Cheng, Valerie Daggett

**Affiliations:** Department of Bioengineering, University of Washington, Seattle WA 98195-5013, USA; E-Mails: chengc2@uw.edu (C.C.); daggett@uw.edu (V.D.)

**Keywords:** amyloid disease, mad cow disease, prion protein misfolding, molecular dynamics, PrP^Sc^ structure

## Abstract

Bovine spongiform encephalopathy (BSE), or mad cow disease, is a fatal neurodegenerative disease that is transmissible to humans and that is currently incurable. BSE is caused by the prion protein (PrP), which adopts two conformers; PrP^C^ is the native innocuous form, which is α-helix rich; and PrP^Sc^ is the β-sheet rich misfolded form, which is infectious and forms neurotoxic species. Acidic pH induces the conversion of PrP^C^ to PrP^Sc^. We have performed molecular dynamics simulations of bovine PrP at various pH regimes. An acidic pH environment induced conformational changes that were not observed in neutral pH simulations. Putative misfolded structures, with nonnative β-strands formed in the flexible *N*-terminal domain, were found in acidic pH simulations. Two distinct pathways were observed for the formation of nonnative β-strands: at low pH, hydrophobic contacts with M129 nucleated the nonnative β-strand; at mid-pH, polar contacts involving Q168 and D178 facilitated the formation of a hairpin at the flexible *N*-terminus. These mid- and low pH simulations capture the process of nonnative β-strand formation, thereby improving our understanding of how PrP^C^ misfolds into the β-sheet rich PrP^Sc^ and how pH factors into the process.

## 1. Introduction

Prion diseases are neurodegenerative diseases that are transmissible, fatal and currently incurable. Such diseases include Creutzfeldt–Jakob disease (CJD) in humans, bovine spongiform encephalopathy (BSE) in cattle, chronic wasting disease in elk and scrapie in sheep [1,2]. In humans, variant CJD is caused by consuming BSE-contaminated products [3,4]. BSE has a significant economic impact on society; in 2003, a single case of BSE in Washington resulted in an estimated annual loss of $7 billion to $10 billion, due to suspended beef exports to overseas markets [5]. 

Prion diseases are caused by the misfolding and aggregation of the prion protein (PrP). The native form of PrP (denoted as PrP^C^) is innocuous and expressed ubiquitously in all mammalian cells. However, PrP^C^ can misfold into PrP^Sc^, which can then self-aggregate and form soluble oligomers that cause neuronal cell death. Misfolding is rare, but it can be induced by an acidic pH environment [6,7,8,9,10]. PrP^C^ is a glycolipid anchored protein expressed at the cell surface. While some PrP^C^ is endocytosed and digested in lysosomes [11], some PrP^C^ is leaked into the cytosol [12]. Therefore, PrP^C^ experiences pH from 7.4 (in cytosol) [13] to 4.6 (in endosome) [14] during its cellular lifetime. The endosome is a site for PrP misfolding [15,16,17,18], and low pH triggers conversion *in vitro* [6,7,8,9,10]. It is therefore of interest to study the pH effects on the native PrP^C^ structure, which ideally would narrow down possible cellular regions, where pH-induced misfolding may occur. The pH-induced structural effect on recombinant PrP has been studied for various species [9,10,19], but not bovine. Recombinant human PrP in between pH 7.2 and five is mainly helical, but PrP misfolds into a β-sheet rich structure at a lower pH range (pH 4 to 3.6) [10].

**Figure 1:**
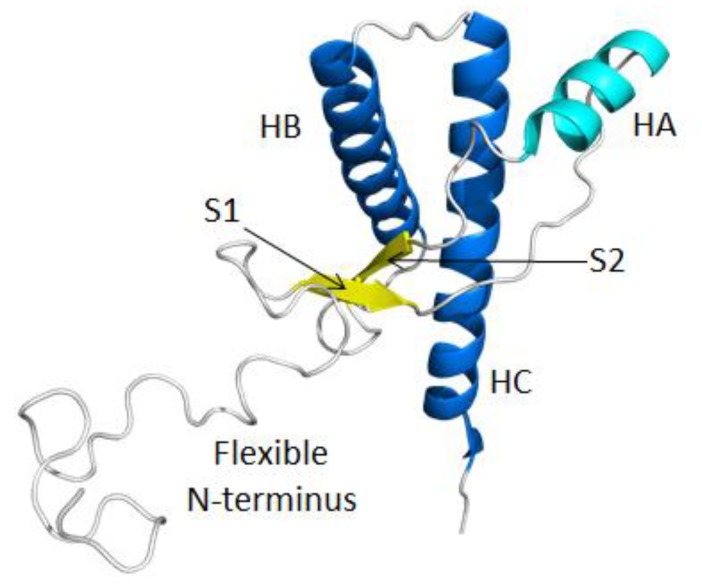
Native structure of bovine PrP^C^ (residues 90–231). HA (Helix A; residues 144–154), HB (residues 173–194) and HC (residues 200–228), S1 (β-strand 1; residues 128–131) and S2 (residues 161–164) are colored in cyan, blue and yellow, respectively. Loop regions are colored in gray. The flexible N-terminal residues 90–127 and C-terminal residues 228–231 were manually constructed. The structure for residues 128–227 was obtained from the bovine PrP NMR structure (Protien Data Bank (PDB) code: 1DWY) [22].

The structure of the C-terminal domain of PrP^C^ has been resolved by NMR methods for various species [20,21,22,23]. The NMR structure of bovine PrP^C^ is almost identical to that of other mammalian species. All mammalian PrP^C^ structures consist of a structured C-terminal domain with three helices (HA, HB and HC), two short β-strands (S1 and S2) and a flexible N-terminal domain (Figure 1). Although no high resolution structure for PrP^Sc^ is available, low resolution experimental methods have suggested that the native PrP^C^ converts from an α-helical rich protein (47% α-helix, 3% β-structure) to the β-sheet rich PrP^Sc^ (43–54% α-helix, 17–30% β-structure) [24,25,26]. The bovine PrP^Sc^ structure is important for understanding the mechanism of cross-species transmission, especially the transmission of BSE to humans [4]. However, the aggregation tendency and heterogeneity of PrP^Sc^ has limited experimental studies to isolate PrP^Sc^ and obtain a high-resolution structure of PrP^Sc^. The cross-species transmissibility of bovine PrP^Sc^ makes experimental characterization of PrP^Sc^ potentially dangerous, and outfitting a lab of a suitable safety level can be a hurdle to perform experimental work. Given the experimental limitations of studying bovine PrP^Sc^, computational methods offer an attractive alternative.

Molecular dynamics (MD) simulations can capture protein dynamics and associated conformational changes at the atomic level. Furthermore, modeling acidic pH environment in simulations is also possible by changing the protonation states of protein amino acids [27,28,29,30]. We have performed acidic pH simulations of various species of PrP [27,28,30,31], including bovine PrP [32]; however, the bovine simulations were not analyzed in depth, and the sampling was limited. In order to more thoroughly investigate the pH-induced misfolding of the bovine PrP, we performed MD simulations of the bovine PrP at neutral, mid- and low pH, which corresponds approximately to ranges around pH 7, five and four, respectively. Five 50 ns simulations were performed for each pH regime, which amounted to a total of 750 ns of simulations. Both mid- and low pH affected the native polar contact network, which resulted in conformational changes of the HA helix and the native sheet. At acidic pH, nonnative β-strands formed, which is a hallmark of prion conversion [27,28,31]. Thus, our MD simulations captured PrP misfolding at acidic pH. The MD-generated misfolded structures are useful for modeling the oligomeric structure of PrP^Sc^ [31,33].

## 2. Results and Discussion

The starting structure of bovine PrP^C^ was constructed from the NMR structure of the C-terminal fragment (PDB code: 1DWY, residues 128–227) [22]. The flexible N-terminal residues 90–127 and C-terminal residues 228–231 were manually constructed, as described previously [27]. We performed simulations of bovine PrP at neutral, mid- and low pH, which corresponded to the approximate pH environment in the cytosol (pH 8–6), endosome (pH ~5–4) and *in vitro* misfolding experiments (pH ~4). Amino acid side chains were protonated differently in order to model the bovine PrP at each pH regime (see the Experimental Section for details). Protonation of side chains had a significant impact on the polar contacts within the protein. At neutral pH, histidines were neutral, while Asp and Glu were negatively charged. At mid-pH, histidines were doubly protonated (positively charged), which allowed them to form salt bridges with other negatively charged amino acids. At low pH, all Asp and Glu acids were protonated (neutral charge). This abolished all salt bridges and significantly disrupted the native hydrogen bonds that involve the side chains of Asp and Glu. Large conformational changes were observed at mid- and low pH compared to the neutral pH simulations. The solvent accessible surface area of hydrophobic regions increased in most of the mid- and low pH simulations. Putative misfolded structures were also observed in both mid- and low pH simulations; the flexible *N*-terminus formed nonnative β-strands by interacting with the S1 strand. Using MD simulations, we have captured the structural effects on bovine PrP induced by acidic pH. These results are discussed in detail below.

### 2.1. Structural Stability and Deviation from the Native Structure

**Figure 2:**
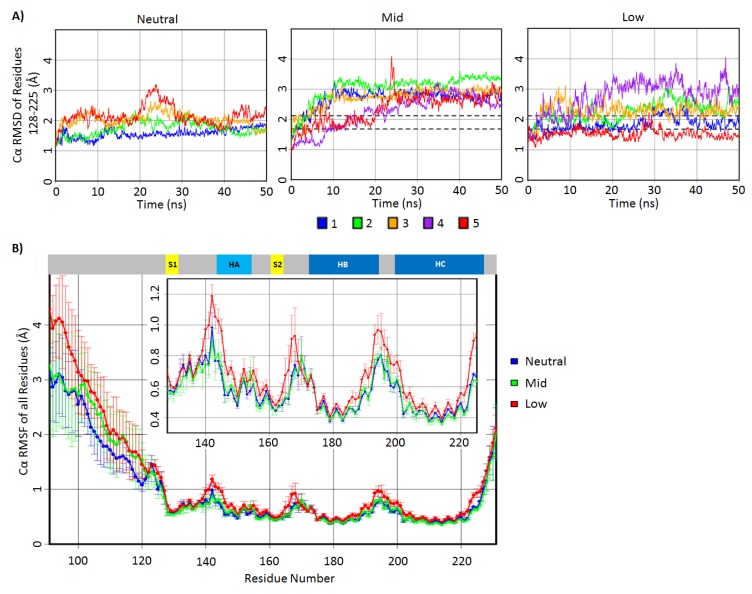
Cα root-mean-square deviation (RMSD) and root mean square fluctuations (RMSF) of the bovine PrP simulations. (**A**) Residue 128–225 Cα RMSD of the neutral, mid- and low pH simulations plotted over time. The area in between the dotted lines is within one standard deviation of the 25–50 ns averaged Cα RMSD of the neutral pH simulations. (**B**) Averaged Cα RMSF plotted for all residues in neutral, mid- and low pH simulations. The inset plot shows Cα RMSF for residues 128–225 in a magnified scale. Native secondary structures of the PrP are shown at the top of the Cα RMSF plots.

To first determine the structural deviations at the C-terminal domain, the Cα root mean square deviation (RMSD) of residues 128–225 was measured for all simulations (Figure 2A). Residues 128–225 include the stable native secondary structures, except for residues 226–231, which are flexible residues at the C-terminus. The Cα RMSD signifies the backbone structural deviation from the native bovine PrP starting structure. The Cα RMSD of residues 128–225 of most simulations reached a plateau by 10 ns, and all simulations reached the steady state after ~25 ns (Figure 2A,B). The average Cα RMSD value over the 25–50 ns interval for all neutral pH simulations was 1.9 Å with a standard deviation of 0.22 Å, indicating small deviations from the native structure. Mid-pH simulations consistently reached higher Cα RMSDs than that of the neutral pH simulations by ~1 Å. The Cα RMSDs in the low pH simulations were less consistent compared to that of the mid-pH simulations. Only low pH Simulations 2 and 4 had higher Cα RMSDs than the neutral pH simulations. These results are consistent with our expectation that acidic pH induces structural changes.

The amount of backbone fluctuations in residues 128–225 are reflected in the Cα root mean square fluctuations (RMSF) for each residue from 128 to 225 (Figure 2B). Generally, the secondary structure elements had lower Cα RMSF (~0.4–0.6 Å) compared to the loop regions (~0.6–1.2 Å). Both neutral and mid-pH had comparable Cα RMSF, with the highest Cα RMSF (0.9 Å) being at residue 140, which is located in the loop preceding HA. Low pH simulations had higher Cα RMSF, especially in the loops. Residues 140–145 around the *N*-terminus of HA were noticeably more mobile (Cα RMSF >0.9 Å) at low pH compared to neutral and mid-pH. 

The Cα RMSD indicate that the C-terminal domain of the mid-pH simulations deviated consistently from the native structure, but it retained a similar backbone flexibility profile to that of the neutral pH simulations. This suggests that the doubly protonated histidines in the mid-pH simulations altered the native PrP conformation, but they did not change the backbone flexibility significantly. The Cα RMSDs of low pH simulations were less consistent, and residues 128–225 were markedly more mobile at loop regions compared to both neutral and mid-pH. The mobility gained in low pH simulations is expected, since all salt bridges and many side chain hydrogen bonds were abolished. 

### 2.2. HA and Native Sheet Conformational Changes

**Figure 3:**
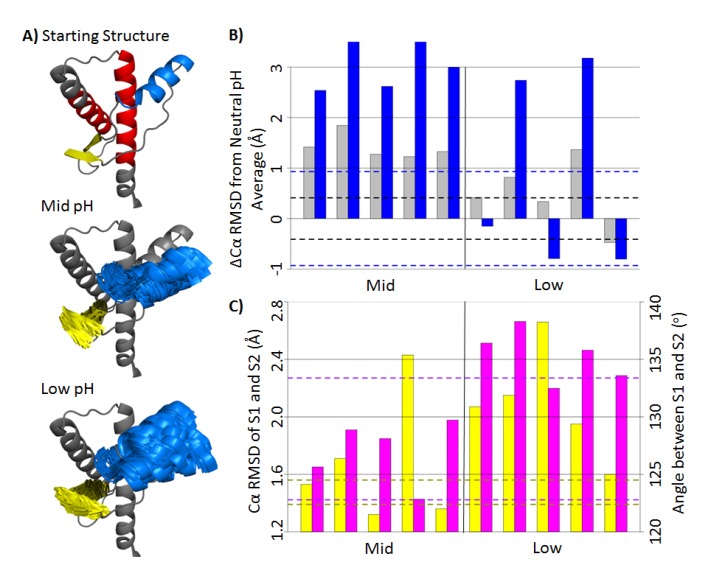
Conformational changes of bovine PrP at mid- and low pH. (**A**) Starting structure of the globular domain (residues 128–228) is shown in gray. HA (residues 144–156) and the native sheet (residues 128–131 and 161–164) are colored as blue and yellow, respectively. Top: the starting structure of the globular domain of the PrP. The stable core region (residues 174–186 and 200–219) is in red. Middle and Bottom: the starting structure of the globular domain overlapped with frames from 25–50 ns of mid- and low pH simulations for HA and the native sheet. The structures are aligned to the stable core region. (**B**) Cα RMSD difference (∆Cα RMSD) from the average value (last 25 ns) of the neutral pH simulations. The ∆Cα RMSD of the globular domain and HA are colored in gray and blue, respectively. The dotted lines indicate Cα RMSD standard deviations of the neutral pH simulations. (**C**) Average Cα RMSD of the native sheet (yellow) and inter-strand angle between S1 and S2 (magenta) for the last 25 ns in each simulation. The area within the yellow dotted lines indicates values within one standard deviation of the averaged Cα RMSD of the neutral pH simulations. Dotted lines in magenta indicate the extreme angles in neutral pH simulations.

To measure tertiary structural deviation from the native structure, a stable core region (residues 174–186 and 200–219) was used as the alignment for the subsequent Cα RMSD measurements. The stable core region is the most well-defined substructure within bovine PrP, as determined by NMR [22]. Cα RMSDs were measured for the globular domain (residues 128–228), HA (residue 144–156) and native strands (residues 128–131 and 161–164) (Figure 3A). For the last 25 ns of the neutral pH simulations, the average Cα RMSD of the globular domain and HA were 2.5 Å and 2.8 Å, respectively. Mid-pH simulations were on average 1.4 Å and 3 Å higher than the neutral pH values for the globular domain and HA, respectively (Figure 3B). Among the low pH simulations, only in Simulations 2 and 4 did the globular domain and HA regions deviate significantly from the native structure. For Simulations 1, 3 and 5, the Cα RMSD of HA were comparable to that of the neutral pH simulations. Therefore, the low pH simulations were able to adopt both native and nonnative conformations of HA, unlike the conformations in mid-pH simulations that were consistently nonnative. The large globular Cα RMSD values correlate with the high Cα RMSD of HA, which suggests that HA is the main contributor to the structural changes in the globular domain. Structures of HA in the last 25 ns of the mid- and low pH simulations are shown in Figure 3A. All mid-pH simulations had HA shifted downward from the native position, while some low pH simulations still retained a native-like HA position. Previous MD simulations of human and hamster PrP [27,28,30,31] and replica exchange simulations of sheep PrP [34] have detected a PrP intermediate state, where HA detaches from HC. Our simulation results suggest that the conformation of HA is sensitive to acidic pH, which is in line with experimental studies on the mouse PrP, where residues 144–149 (a portion of HA) increase in mobility at acidic pH [34].

The average Cα RMSDs of the β-sheet in the neutral, mid-­ and low pH simulations were 1.48 Å, 1.67 Å and 2.09 Å, respectively. In addition to the Cα RMSD, the inter-strand angles of the native sheets were measured to identify different native sheet conformations (Figure 3C). The inter-strand angles were defined, such that they were in between 0^o^ (flat and parallel β-strands) and 180^o^ (flat and anti-parallel β-strands). The average inter-strand angles and native sheet Cα RMSDs for the last 25 ns of mid- and low pH simulations are shown in Figure 3C. All low pH simulations had average inter-strand angles >132^o^, whereas all mid-pH simulations had average inter-strand angles <129^o^. For neutral pH simulations, the inter-strand angles were between 123^o^ and 133^o^, which falls in the range of both mid- and low pH inter-strand angles. Although the inter-strand angles at neutral pH were not consistent across simulations, native strands in the mid-pH simulations were consistently more twisted compared to the low pH simulations. Conformational changes of the native strand S2 is expected, because the structural stability of S2 is sensitive to acidic pH [34]. A PrP^Sc^-specific antibody that binds to the “YYR epitope” in S2 suggests that S2 undergoes conformational changes upon misfolding [35]. Therefore, the conformational differences of the native sheet in mid- and low pH simulations may be related to the misfolding of PrP. 

### 2.3. Structural Changes and Alterations in Native Polar Contacts

In order to understand the cause of the deviations of HA and native strands from their native conformation at both mid- and low pH, the polar contacts near HA and the native strands were analyzed (Table 1). Polar contacts include both hydrogen bonds and salt bridges between residues (see the Experimental Section for details). Mid-pH simulations were able to form nonnative salt bridges with the doubly protonated histidines. Most native side chain polar contacts were lost at low pH. These changes in polar contacts led to the detachment of HA and alterations to the β-sheet, as well as other changes in the protein. These issues are discussed in detail in the following sections.

**Table 1:** Population (in percent of time) for the last 25 ns of polar contacts between pairs of residues in mid- and low pH simulations and the average values for neural pH simulations. All polar contacts listed are between residue side chains, except for |Y162-T183, where the side chain of T183 formed a hydrogen bond with the backbone amide of Y162.

pH	Simulation	HA relevant contacts					S2 relevant contacts			
		Y149-D202	R156-D202	E146-R208	K194-E196	H155-E196	Y162-T183	Y162-E186	Y163-E221	E186-H187
Neutral	Average	90.7	51.4	73.7	52.8	0.0	29.7	28.1	70.9	0.0
Mid	1	0.0	100.0	100.0	100.0	100.0	20.1	29.3	99.9	0.0
	2	0.3	100.0	100.0	3.9	100.0	2.6	100.0	99.2	89.3
	3	0.0	100.0	100.0	0.0	0.0	30.7	15.0	66.5	0.0
	4	0.0	100.0	100.0	0.3	7.2	0.5	94.2	99.3	0.0
	5	0.0	16.2	0.0	70.1	0.0	0.5	48.2	100.0	0.0
Low	1	0.0	0.0	0.0	0.0	0.0	84.4	0.0	2.9	0.0
	2	0.1	0.0	0.0	0.0	0.0	74.6	0.0	1.0	0.0
	3	0.0	0.0	0.0	0.0	0.0	0.2	0.0	1.2	0.0
	4	0.0	0.0	0.0	0.0	0.0	57.1	0.0	0.0	0.0
	5	0.0	0.0	0.0	0.0	0.0	66.4	0.1	0.5	0.0

#### 2.3.1. Polar Contacts with HA

**Figure 4:**
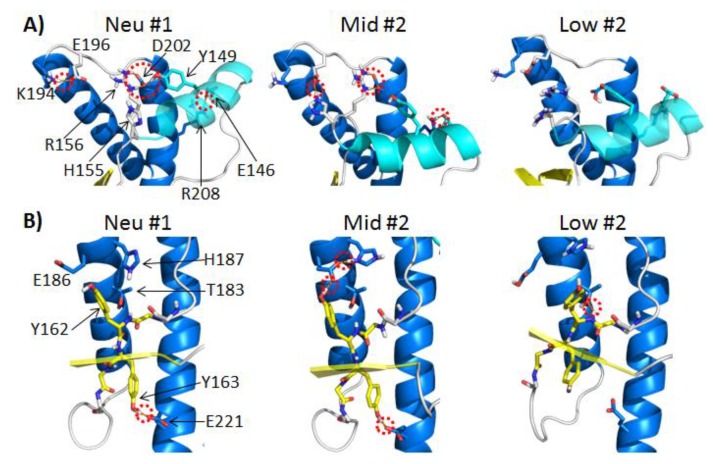
Changes in polar contacts at different pH. Structures from neutral pH Simulation 1, mid-pH Simulation 2 and low pH Simulation 2 at 30 ns are shown in (A) and (B). Red dotted circles indicate polar contacts. Relevant polar residues are shown as sticks. (**A**) Relevant polar contacts around HA, where HA is colored in cyan. (**B**) Relevant polar contacts around S2, where the backbone of S2 is shown as sticks with carbon atoms colored in yellow.

In the neutral pH simulations, the populated polar contacts (average occupancy >50%) around HA were: Y149-D202, R156-D202, E146-R208 and K194-E196 (Figure 4A). HA was anchored to HC by the side chain polar contacts, Y149-D202 and E146-R208. The salt bridge R156-D202 tethered the C-terminus of HA to HC. Among these three HA stabilizing contacts, the hydrogen bond, Y149-D202, was the most stable contact, and on average, it was populated 91% of the time in the neutral pH simulations (Table 1). In the mid pH simulations, only two of these HA stabilizing contacts were preserved: E146-R208 and R156-D202. The hydrogen bond between Y149-D202 was completely lost in all mid-pH simulations. The doubly protonated H155 formed a nonnative salt bridge with E196. The salt bridge H155-E196 was 100% populated in two of the mid pH simulations. These changes in the polar contacts around HA were accompanied with the large deviation of HA from its native position (Figure 3B). 

Large HA movements were also observed in low pH Simulations 2 and 4 (Figure 3B). The low pH simulations lost all of the polar contacts around HA (percentage of time in contact during 25–50 ns, <1%). While both-mid and low pH displayed large deviations of HA from its native position, HA in the mid-pH simulations was less flexible compared to HA at low pH, as indicated by the Cα RMSF (Figure 2B). This suggests that the polar contacts at mid-pH have a stabilizing effect on the nonnative position of HA. These observations are consistent with previous NMR studies on the human PrP at acidic pH, which suggest that the doubly protonated H155 has an impact on the PrP structure [36]. This was shown explicitly in two of our mid pH simulations, where H155 formed a nonnative salt bridge with E196, stabilizing the nonnative position of HA. As for low pH simulations, the loss of all salt bridges and many side chain hydrogen bonds allowed more flexibility at HA, as indicated by Cα RMSF (Figure 2B). These results are in agreement with previous MD simulations of the HA region of PrP, where Lingenheil *et al*. suggest that electrostatic interactions are crucial to the stability of HA [37].

#### 2.3.2. Polar Contacts with S2

In the neutral pH simulations, three polar contacts, Y163-E221, Y162-T183 and Y162-E186, had average occupancies of 71%, 30% and 28%, respectively (Table 1). These contacts anchored S2 to the stable core region of PrP (Figure 4B). In most of the mid-pH simulations, the hydrogen bond, Y162-T183, was insignificant (<30% occupancy), but the Y162 and Y163 side chains both formed relatively stable hydrogen bonds with E186 and E221, respectively. In mid-pH Simulation 2, the side chain conformation of E186 was further stabilized by forming a nonnative salt bridge with the doubly protonated H187 (Figure 4B). This interaction indirectly stabilized the hydrogen bond between Y162 and E186 (100% occupancy).

The hydrogen bonds, Y162-E186 and Y163-E221, anchored S2 to HB and HC. These two anchors favored a more twisted inter-strand angle, as observed in mid-pH simulations (Figure 3C). In low pH simulations, hydrogen bonds Y162-E186 and Y163-E221 were lost, due to the protonation of the Glu acids, but there was a gain in the hydrogen bond, Y162-T183, in most low pH simulations. In low pH Simulation 1, hydrogen bond Y162-T183 was occupied 84% of the time, which is significantly higher than that of any neutral and mid-pH simulations. The increase in occupancy for this hydrogen bond, Y162-T183, was also observed in previous MD simulations of human PrP at low pH [38]. The native strand, S2, lost the two anchors at low pH, which allowed S2 to pack closer to HB. This favored the formation of the hydrogen bond, Y162-T183. With the loss of the two anchors, the native strands became less twisted, as indicated by inter-strand angles (Figure 3C). Given that the exposed native strand, S1, is at the packing interface between PrP dimers from sheep PrP crystals [39], changes in the native sheet region could modulate the intermolecular interactions between PrP. The native strand, S1, is also known for recruiting nonnative β-strands from the flexible *N*-terminus, as shown in previous misfolded structures identified from MD simulations [27,28,30,31,38,40]. Changes of the inter-strand angle might also have an effect on the formation of nonnative β-strand, which is an important hallmark for misfolding.

### 2.4. Solvent Exposure of Hydrophobic Regions

**Figure 5:**
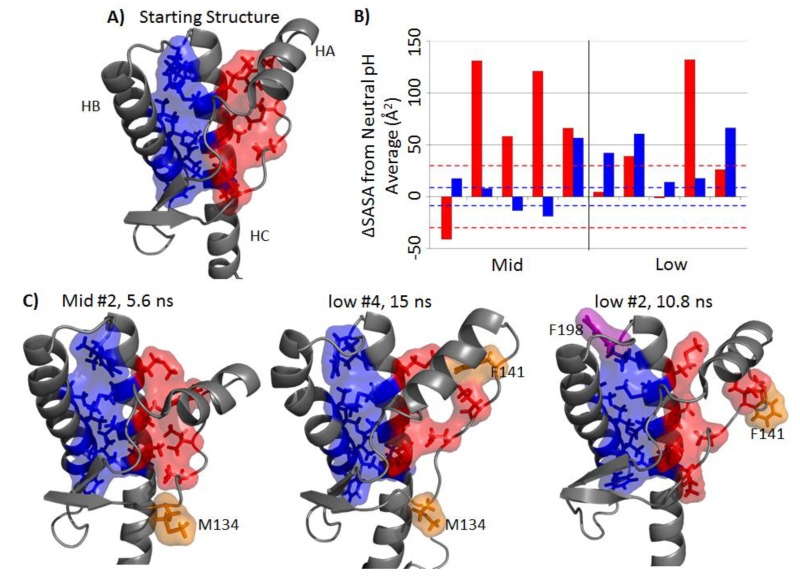
Disruption of the hydrophobic core. (**A**) Two regions of the hydrophobic core: HA-HC (red) includes residues 134, 137, 139, 141, 205, 209 and 213; HB-HC (blue) includes residues 175, 176, 179, 180, 184, 198, 203, 206, 210, 214 and 215. (**B**) Side chain solvent accessible surface area (SASA) deviation from the averaged neutral pH simulation for the last 25 ns, where red and blue bars corresponds to the HA-HC and HB-HC hydrophobic core region, respectively. The area within a pair of dotted lines indicates values within one standard deviation of the averaged SASA of the neutral pH simulations. (**C**) Typical structures where significant SASA increased for mid- and low pH simulations. Magenta and orange residues are residues that become significantly exposed in the HB-HC region and the HA-HC region, respectively.

The hydrophobic core of PrP consists of a set of hydrophobic residues within the globular domain. There are two regions of the hydrophobic core: the HA-HC region and the HB-HC region (Figure 5A). The average solvent accessible surface areas (SASA) for hydrophobic regions HA-HC and HB-HC were 88 Å^2^ and 191 Å^2^, respectively. The difference in SASA with respect to the neutral pH simulations are shown in Figure 5B. The HA-HC hydrophobic core in the mid-pH simulations was significantly exposed in four out of five simulations (Figure 5B). As for the HB-HC portion, only mid-pH Simulation 5 had a large difference in SASA with respect to the neutral pH simulations (57 Å^2^). Among low pH simulations, only Simulation 4 had the HA-HC hydrophobic core significantly exposed. Low pH Simulations 1, 2 and 5 tended to have a larger HB-HC hydrophobic core SASA than that of the average HB-HC hydrophobic core SASA in neutral pH simulations (>42 Å^2^ difference). Residues that contributed significantly to the increased solvent exposure were M134, F141 and F198, where both M134 and F141 are part of the HA-HC hydrophobic core and F198 is in the HB-HC hydrophobic core (Figure 5C).

NMR studies of human PrP have shown that HA becomes less stable at acidic pH [36]. This is in agreement with the disrupted hydrophobic packing and detachment between HA and HC in the mid-and low pH simulations. The hydrophobic residue, F141, is one of the less stable residues, as identified by unfolding experiments of bovine PrP [41]. This was reflected in our simulations, where F141 detached from the hydrophobic core and became solvent exposed at low pH. Previous MD simulations have shown that large displacement of HA can be caused by mutations that disrupt the hydrophobic packing between HA and HC [42]. In addition, detachment of HA from HC is necessary for fibril formation, as indicated in *in vitro* [43,44] and *in vivo* [43] experiments. This suggests that the repositioning of HA along with the exposure of the HA-HC hydrophobic region contribute to misfolding.

In the starting structure, F198 was buried in between HB and HC, with less than 20 Å^2^ SASA of the side chain exposed. In the low pH simulations, the HB-HC loop became highly flexible (Figure 2B), and the side chain of F198 became solvent exposed (~50–100 Å^2^). The change in flexibility of the HB-HC loop and the increased solvent exposure of F198 side chain might be related to the misfolded conformation of residues 187–206 detected by a PrP^Sc^-specific antibody [45]. The F198S human pathogenic mutation causes a void in the HB-HC hydrophobic core and reduces the thermodynamic stability of PrP, which, in turn, increases the propensity of PrP^Sc^ conversion [46,47]. This suggests that the loss of hydrophobic packing with F198 facilitates misfolding. 

### 2.5. Formation of Nonnative β-Strands

An increase in β-sheet content is evident in the process of PrP^C^ misfolding to PrP^Sc^ [24,25,26], and therefore, it is expected that misfolding involves the formation of nonnative β-strands. Previous MD simulations have indicated that the flexible *N*-terminus can form nonnative strands, and they are the putative aggregation sites for PrP^Sc^ oligomerization [31,33]. Although misfolding is rare, some of our simulations captured the misfolding process. Nonnative strands were formed in low pH Simulation 3 and mid-pH Simulation 2. In both simulations, the native strand, S1, served as the nucleation site for recruiting the nonnative strands from the flexible *N*-terminus. The process of nonnative β-strand formation is described in detail below.

#### 2.5.1. Hydrophobic Contacts at Low pH

**Figure 6:**
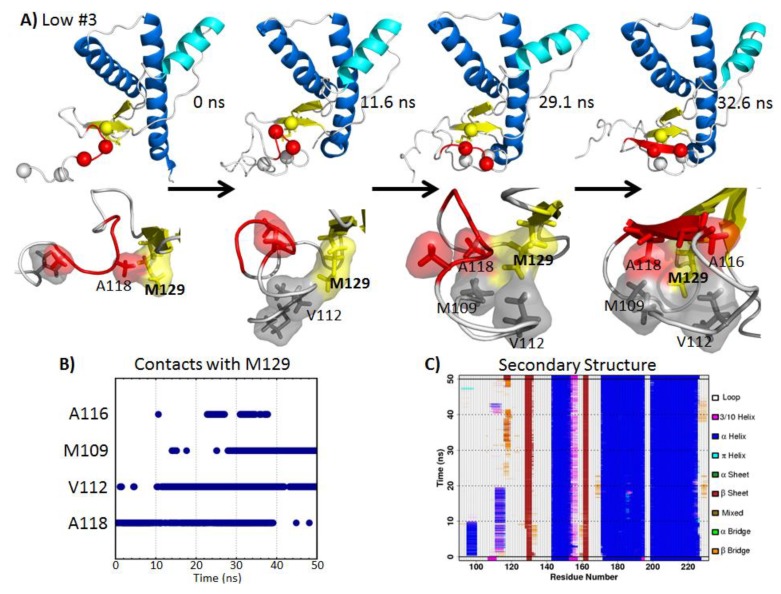
Illustration of nonnative β-strand formation at the *N*-terminus facilitated by *N*-terminal hydrophobic contacts with M129 in low pH Simulation 3. (**A**) The top panel shows the bovine PrP, with important hydrophobic residues indicated by spheres. Residues in red participate in the nonnative β-strand. The lower panel shows the close-up view of M129 and the neighboring residues. (**B**) Hydrophobic contacts with M129 over time. (**C**) PrP secondary structure, where orange and red regions indicate β-structures. A nonnative strand was formed from ~30–42 ns.

In low pH Simulation 3, a nonnative strand formed at the flexible *N*-terminus, where residues 116-120 docked to the native strand, S1 (Figure 6A). Hydrophobic contacts between M129 and residues at the *N*-terminus facilitated the nucleation of the nonnative strand (Figure 6B). The starting structure of the *N*-terminus was extended away from the globular domain, and only A118 and M129 were in contact. After 11.6 ns, the *N*-terminus collapsed, and V112 was in contact with M129. At 29.1 ns, residues A118, V112 and M109 formed a hydrophobic cluster around M129. At this point, a β-bridge was already formed between the nonnative strand region and S1 (Figure 6C). Eventually, a β-strand formed at 32.6 ns, with A116 participating in the hydrophobic cluster around M129. This β-strand was stable for approximately 10 ns (Figure 6C). 

The process of forming the nonnative β-strand was driven by hydrophobic contacts with M129. Prion protective polymorphisms in both human [48] and cervid [49,50] PrP at residue 129 are found in nature, which suggests that residue 129 affects the susceptibility to prion diseases in some species, and Chen *et al.*, 2014, addresses the differences between M129 and V129 in human PrP simulations [51]. Hydrophobic residues M109, V112, A116 and A118 make contact with M129 and are part of the neurotoxic peptide, PrP 106–126 [52,53,54,55], which becomes enriched in β-structure at acidic pH [53,54]. These experimental observations are in line with our low pH simulation result, where residues 116–120 formed a nonnative β-strand.

#### 2.5.2. Polar Contacts at Mid-pH

**Figure 7:**
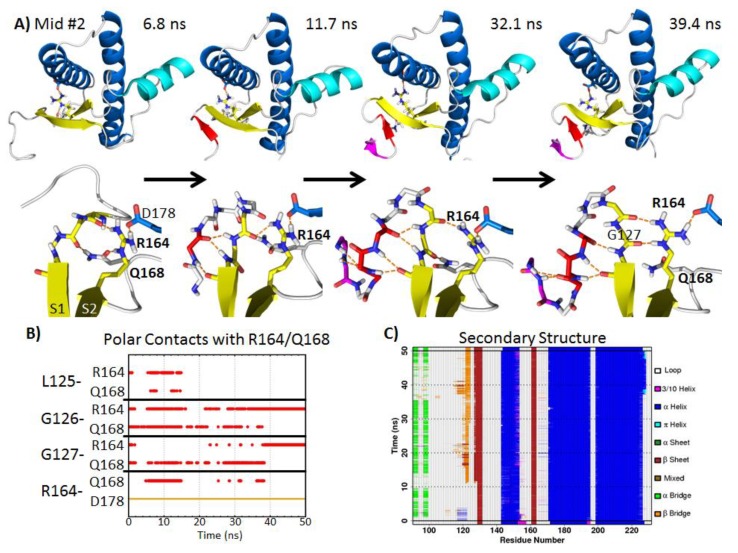
Illustration of nonnative strand formation at the *N*-terminus facilitated by *N*-terminal polar contacts with side chains of R164 and Q168 in mid-pH Simulation 2. (**A**) The top panel shows the PrP construct with the nonnative strands colored in red and magenta. Side chains of R164, Q168 and D178 are shown as sticks. The lower panel shows the close-up view of R164, Q168 and relevant *N*-terminal residues. (**B**) Hydrogen bonds and salt bridge formed with R164 or Q168 over time. (**C**) Analysis of PrP secondary structure, where orange and red regions indicate β-structures. A nonnative strand was formed from ~11.7–50 ns.

In mid-pH Simulation 2, R164 and Q168 formed hydrogen bonds with the backbone carbonyls of residues 125–127 near the *N*-terminus of S1 (Figure 7A,B). This oriented the backbone amides of residues 125–127 away from the globular domain of the PrP. This backbone conformation favored the formation of a hairpin turn, which was observed after 11.7 ns in simulation (Figure 7A). Residues 121–123 formed stable β-bridges with residues 126 and 127 (Figure 7C). At 32.1 ns, residues 118–119 formed another pair of β-bridges with residue 121. The hairpin turn residues 123–127 at the *N*-terminus of S1 had a similar conformation compared to the turn residues of α-hairpins found in previous MD simulations of the PrP [38] and polyglutamine peptides [56]. α-sheet structures represent putative toxic conformers in amyloid disease [57]. The terminal turn residues in an α-hairpin have α_L_ conformations [56]. In this mid-pH simulation, the α_L_ backbone conformation at residue 127 was stabilized by backbone hydrogen bonds with the side chains of R164 and Q168. At 39.4 ns, Q168 lost its hydrogen bond with residue 126. Instead, the R164 side chain formed two hydrogen bonds with the backbone carbonyls of residues 126 and 127. This pair of side chain-main chain hydrogen bonds also stabilized the α_L_ conformation at residue 127, thereby stabilizing the hairpin turn. Furthermore, the side chain conformation of R164 was stabilized by forming a salt bridge with D178 (occupied throughout the entire simulation).

Residues 168 and 178 are related to prion-susceptible polymorphisms [58] and a human PrP pathogenic mutation [59], respectively. Previous MD simulations have shown that the loss of the native salt bridge, R164-D178, caused by the D178N mutation, does not significantly disrupt the structured C-terminal domain of the PrP [60]. The R164-D178 salt bridge, however, was able to affect the flexible *N*-terminus structure in mid-pH Simulation 2. The stable salt bridge R164-D178 placed the guanidinium group of R164 in the same plane as S1. This favored the interaction between the side chain of R164 and the backbone carbonyl of G126 and G127, which encouraged the hairpin formation at the flexible *N*-terminus. As for Q168, its side chain formed hydrogen bonds with the hairpin turn residues, G126 and G127. Since polymorphism at residue 168 modulates the prion susceptibility of sheep [58] and the mouse PrP mutation, Q168R, completely abolishes PrP^Sc^ formation [61], we hypothesize that Q168 plays an important role in the mechanism of misfolding.

The biological relevance of residues 168 and 178 supports our computational results; both residues 168 and 178 facilitated the nonnative strand formation at the flexible *N*-terminus. Residues 168 and 178 stabilized the α_L_ conformation at residue 127, facilitating formation of a new hairpin. This resulted in an increase in β-sheet content, which is a hallmark of conversion.

## 3. Experimental Section

The starting structure of the simulations was derived from the NMR structure of bovine PrP obtained at pH 4.5 (PDB code: 1DWY [22]). The missing regions, the flexible *N*-terminus (residues 90–127) and the *C*-terminus (residues 228–231), were manually constructed, as described previously [27]. It is important to include the *N*-terminal fragment, because there is strong evidence that it plays an important role in misfolding [62,63,64,65]. Briefly, residues 90–127 were constructed using limited Nuclear Overhauser Effect (NOE) restraints from the hamster PrP for the same region. The flexible *N*-terminus was built such that it lies approximately perpendicular to S1 and S2. The *N*-terminus was extended away from the globular domain, to minimize interactions that could bias simulations. The C-terminal residues 228–231 were manually built by extending the C-terminus of HC. 

The pH environment of the simulation was modeled by altering the protonation state of amino acids. At neutral pH, all Glu and Asp were negatively charged (unprotonated). H144 was δ-protonated, whereas all other histidines were ε-protonated, following the results of Langella *et al.* [66]. Langella *et al*. have estimated the pKa values of the human PrP. Their results show that H140 prefers the δ tautomeric form, whereas all other histidines preferred the ε tautomeric form at neutral pH. At mid-pH, all histidines were doubly protonated (positive charge). Asp and Glu remained negatively charged. Since the lowest pKa among histidine residues in PrP is ~5.5 [66] and the pKa of Glu and Asp acids are ~4, the mid-pH environment in simulation roughly corresponds to pH 5. At low pH, all Glu and Asp residues were protonated, and histidines were doubly protonated. Therefore, the simulated pH environment should be lower than that of the pKa values of Glu and Asp residues (pKa ~4).

MD simulations were performed at 298 K using *in lucem* molecular mechanics (*il*mm) [67]. The NVE microcanonical ensemble (constant number of atoms, volume and total energy) was employed. The Levitt *et al.* force field [68] was employed for the protein. Preparation of the simulation followed previously described protocols [69]. Briefly, the steepest descent minimization was performed for the starting structure *in vacuo* for 1,000 steps. The minimized structure was placed in an empty rectangular water box with the following dimensions: 88.7 Å by 77.1 Å by 48.1 Å. The system contained 10,100 water molecules, and the box volume was set to reproduce the density of water at 298 K (0.997 g/mL [70]). The F3C water model [71] was implemented. The walls of the periodic box were at least 10 Å away from the protein. Water molecules were minimized for 1,000 steps. After that, 1 ps of water dynamics was performed to heat the system to 298 K. Water molecules were minimized for another 500 steps. The protein was then minimized for 500 steps. For every two steps, a force-shifted non-bonded cutoff of 10 Å was updated [72]. A time step of 2 fs was used for performing MD simulations. Five 50 ns simulations were performed at neutral, mid- and low pH each, which in total amounted to 750 ns. Random number seeds were used to randomize the initial velocities for atoms in different simulation replicates.

*il*mm [67] was used to perform Cα RMSD, DSSP (Define Secondary Structure of Proteins) [73] and SASA [74] analyses at 10 ps granularity. The Cα RMSF was measured using 1 ns windows throughout the simulations and averaged across all five simulations for each pH regime. Native sheet angles were measured at 100 ps granularity. Briefly, a vector was used to fit the Cα atoms on each native strand (residues 128–131 for S1 and 161–164 for S2). The angle between the two vectors were calculated using Visual Molecular Dynamics (VMD) [75]. Atomic contacts were classified as salt bridges (N-O distances ≤4.6 Å between oppositely charged residues), hydrogen bonds (H-acceptor distance ≤2.6 Å and donor-H-acceptor angle >135^o^) and hydrophobic contacts (C-C distance ≤5.4 Å). Polar contacts include both salt bridges and hydrogen bonds formed between atoms. For the neutral pH simulations, the averaged values are over four simulations. This is because neutral pH Simulation 4 had a large conformational change at HA (average HA Cα RMSD = 8 Å). Due to the significant deviation from the native structure, neutral pH Simulation 4 was considered an outlier.

## 4. Conclusions

We have identified common misfolding events of bovine PrP at mid- and low pH, such as: (1) detachment of HA from HC; (2) an increase in solvent exposure of the hydrophobic core; and (3) the formation of nonnative strands in the flexible *N*-terminus. The HB and HC helices remained largely intact, in agreement with the experiment [76]. Although putative misfolded structures in both the mid- and low pH simulations had nonnative strands in the flexible *N*-terminus, the mechanisms for their formation were significantly different. At mid-pH, a network of polar contacts involving the Q168 and D178 side chains stabilized the hairpin turn at the *N*-terminus of S1, which favored the formation of a stable nonnative strand at the flexible *N*-terminus. At low pH, M129 formed hydrophobic contacts with residues in the *N*-terminus, which facilitated the formation of a nonnative strand. The presence of different misfolding pathways of bovine PrP is consistent with the heterogeneous nature of PrP^Sc^. The key residues involved in the misfolding are single-nucleotide polymorphisms associated with disease susceptibility (M129, Q168) and the site of a pathogenic mutation (D178N), which supports the biological relevance of our results. These simulations provide further insight into how PrP^C^ misfolds into a β-sheet rich structure under acidic pH conditions. Furthermore, the identified misfolded structures may help with the construction of oligomeric bovine PrP^Sc^ models and provide much-needed clues to understand the molecular mechanism of disease transmission. 
